# Perceived social support and psychological flexibility mediate the relationship between positive solitude and psychological wellbeing among Turkish adults

**DOI:** 10.3389/fpsyg.2026.1714036

**Published:** 2026-04-08

**Authors:** Murat Yıldırım, Gülçin Güler-Öztekin, Orhan Koçak, Samet Makas, Abdulmohsen Mohammed Abdullah Alkhulayfi

**Affiliations:** 1 Department of Psychology, Faculty of Science and Letters, Agri Ibrahim Cecen University, Ağrı, Türkiye; 2 Psychology Research Center, Khazar University, Baku, Azerbaijan; 3 Department of Social Work, Faculty of Health Sciences, Istanbul University-Cerrahpasa, Istanbul, Türkiye; 4 Department of Guidance and Psychological Counselling, Faculty of Education, Sakarya University, Sakarya, Türkiye; 5 Department of Business Administration, Faculty of Economics and Administration, King Abdulaziz University, Jeddah, Saudi Arabia

**Keywords:** perceived social support, positive solitude, psychological flexibility, psychological wellbeing, Turkish adults

## Abstract

**Introduction:**

This study is the first to examine the mediating roles of perceived social support and psychological flexibility in the relationship between positive solitude and psychological wellbeing among Turkish adults.

**Methods:**

A total of 1,060 participants (69.25% female), aged 18 to 61 years (*M* = 24.11, *SD* = 6.84), took part to complete the Positive Solitude Scale, Brief Perceived Social Support Questionnaire, Psy-Flex Scale, and Riverside Eudaimonia Scale.

**Results:**

Findings revealed that positive solitude significantly predicted perceived social support, psychological flexibility, and psychological wellbeing. Both perceived social support and psychological flexibility had significant effects on psychological wellbeing and mediated the association between positive solitude and psychological well being.

**Discussion:**

These results suggest the important roles of perceived social support and psychological flexibility in related to the effect of positive solitude on psychological wellbeing.

## Introduction

With the emergence of positive psychology, there has been a growing interest in exploring the positive aspects of human life, shifting the focus from merely treating mental illness and dysfunction to understanding and associated with human strengths, wellbeing, and flourishing (Seligman and Csikszentmihalyi, 2000). Psychological wellbeing is a multidimensional concept that includes the overall satisfaction and optimum functioning experienced by individuals in their lives. Studies addressing this concept have focused on two main perspectives. One of these is the hedonic approach. This suggests that wellbeing includes happiness, pleasure, general satisfaction with life, and the avoidance of negative emotions. The other is the eudaimonic approach. This view posits that wellbeing is related to finding meaning in life, the individual’s self-actualisation, and development (Ryan and Deci, 2001). This study focuses on eudaimonic wellbeing, which encompasses the individual’s development.

Existing literature has shown that psychological wellbeing was effective in individuals’ self-acceptance, establishing positive relationships, providing autonomy, personal development and creating a life purpose (Morales-Rodríguez et al., 2020). Individuals with higher psychological wellbeing were less likely to experience psychological distress (Baroni et al., 2018). These individuals were more resilient because they used more effective coping strategies for the problems they encountered (Foster et al., 2020). Psychologically well individuals were more conscious of behavioural addictions (Ponnusamy et al., 2020). Therefore, identifying the factors that contribute to psychological wellbeing is of great importance for supporting individuals in many aspects.

Positive solitude refers to a voluntary positive experience of being alone that people can enjoy. While physical solitude is an objective state, loneliness is a negative perception of this state, and positive solitude is a phase of inner empowerment (Palgi et al., 2021). Emotion regulation through music listening and mindfulness contributed to positive solitude (Bachman et al., 2022). When individuals actively choose to be alone, the experience of solitude helps them relax and reduce stress (Nguyen et al., 2018). Experiencing awe that made people feel alone but not lonely led to positive attitudes toward solitude. These experiences served as a time for self-reflection and spiritual awakening (Yin et al., 2024), which are necessary for individuals’ wellbeing and self-actualisation (Harrington and Loffredo, 2010).

The relationship between positive solitude and psychological wellbeing is explained by incorporating the concepts of autonomy and inner awareness into a theoretical framework grounded in Self-Determination Theory (SDT). According to SDT, autonomous motivation is defined as wanting to do something because you enjoy it and/or because it is valuable (Ryan and Deci, 2000). Ryan and Deci (2000) argued that significant individual differences in the need for autonomy are rare because achieving autonomy is a fundamental developmental task for everyone. In this context, we can define positive solitude as a state in which the individual’s fundamental developmental need for autonomy is fulfilled. Research shows a positive relationship between autonomy and psychological wellbeing (Bülow et al., 2022; De-Juanas et al., 2020). Based on all this, it can be stated that positive solitude, which also provides a space where autonomy needs are met, will have a positive effect on the individual’s wellbeing.

Studies investigating the link between positive solitude and wellbeing have shown that positive solitude contributes to many positive effects on positive functioning, including greater positive affect (Toyoshima and Sato, 2019), personal growth and a vibrant inner life (Smith et al., 2023). Positive solitude provided individuals with greater autonomy satisfaction in a voluntary, authentic, and externally pressure-free manner, while also reducing stress levels. These benefits had a cumulative effect; that is, spending time alone by one’s own choice strengthens an inner sense of freedom and contributes to the process of self-discovery (Weinstein et al., 2023). These findings reveal that individuals’ conscious experience of solitude can be an important resource for personal development, psychological health and wellbeing.

## Mediating roles of perceived social support and psychological flexibility

People are social beings by nature, and they build strong bonds, especially with those they feel close to, trust, and spend time with. In this regard, they share both positive experiences (success, happiness, enjoyable moments) and negative experiences (stress, sadness, difficulties) through their social networks (Reis et al., 2010). Individuals need social support in this sharing process. Perceived social support refers to how individuals evaluate the support offered to them and what they think about this support (Shahry et al., 2016). Many studies have shown that adequacy of social support was associated with fewer psychological problems (Bedaso et al., 2021), better physical health (Uchino et al., 2018) and better academic life (Luan et al., 2023). Social support was negatively associated with the experience of negative solitude and positively associated with coping strategies (Hussin et al., 2021). In addition, numerous studies have asserted that social support contributes to subjective wellbeing (Yıldırım and Tanrıverdi, 2021), psychological wellbeing (Nagy-Pénzes et al., 2020) and social wellbeing (Yu et al., 2021) of individuals.

Previous research has also demonstrated that perceived social support mediated the associations regarding wellbeing. Social support reduced the deleterious effects of burnout on psychological wellbeing (Rehman et al., 2020). Colleagues and supervisors mediated the link between job stress and mental wellbeing (Mensah, 2021). Social support served as a protective resource, preventing substance use behaviour (Arslan, 2024). In addition, receiving help, guidance and information support from social contacts mitigated the positive relationship between loneliness and anxiety, depression, and somatic symptoms (Hutten et al., 2021). These studies emphasise that social support has both a protective and an increasing role.

Psychological flexibility refers to the ability to consciously experience the present moment. When individuals focus on valued ends, they persist or change their behaviour (Hayes et al., 2011). Psychological flexibility led to several positive psychological outcomes, such as high psychological wellbeing (Guerrini Usubini et al., 2021), low psychological distress (Masuda and Tully, 2012), a more meaningful life (Arslan and Allen, 2022), and low loneliness (Frinking et al., 2020). Psychological flexibility supported adaptive responses in coping with difficult situations and helped maintain mental health (Dawson and Golijani-Moghaddam, 2020).

There are various studies in the literature that emphasise the mediating role of psychological flexibility. Psychological flexibility mediated the relationship between resilience and distress, mental and physical health, and quality of life (Pakenham et al., 2024). Psychological flexibility acts as a mediator in the relationship between health anxiety and mental health (Landi et al., 2020). Psychological flexibility strengthened the relationship between emotional intelligence and psychological wellbeing (Semiromi et al., 2023). These results show that psychological flexibility has a protective effect on mental health problems and a strengthening effect on wellbeing.

Like every period of individuals’ lives, adulthood has a dynamic structure in which their wellbeing changes over time and fluctuates internally (Sonnentag, 2015). Therefore, it is essential to determine the factors that contribute to and strengthen the wellbeing of adults. The above-mentioned studies show that positive solitude is positively associated with psychological wellbeing. Perceived social support and psychological flexibility may have a strengthening effect on this relationship. However, to our knowledge, there is no study examining the mediating roles of perceived social support and psychological flexibility in the relationship between positive solitude and psychological wellbeing among adults. Therefore, we aimed to examine this effect in this study.

## Present study

**Figure 1:**
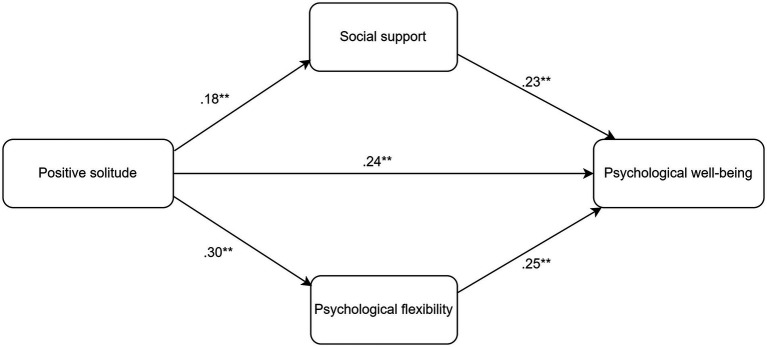
Structural model illustrating the associations between the variables (**p < 0.001).

This study examined the potential mediating roles of perceived social support and psychological flexibility in the relationship between positive solitude and psychological wellbeing. Three hypotheses were established: (1) positive solitude will have a positive association with perceived social support, psychological flexibility, and psychological wellbeing; (2) both perceived social support and psychological flexibility will be positively related to psychological wellbeing; and (3) perceived social support and psychological flexibility will mediate the association between positive solitude and psychological wellbeing. The conceptual model is illustrated in Figure 1.

## Method

### Participants

The sample of this cross-sectional study comprised 1,060 Turkish adults whose ages ranged between 18 and 61 years (*M* = 24.11, *SD* = 6.84). The sample consisted of 69.25% females and 30.75% males. Regarding perceived economic status, the majority (78.00%) considered themselves of average economic standing, while 13.10% rated their status as below average, and 8.90% as above average. Most participants were single (73.30%), followed by 25.10% who were married, and 1.60% who were widowed or divorced.

### Measures

#### Positive solitude

Positive solitude was measured with the Positive Solitude Scale (PSS) (Palgi et al., 2021), which measures an individual’s attitude and preference for positive solitude. The PSS includes 9 items on a single dimension, rated on a 5-point Likert scale ranging from 1 (not at all) to 5 (most of the time). The total score is the sum of all item scores, with higher scores reflecting a more positive attitude toward and preference for solitude. The Turkish adaptation and validation of the scale were conducted by Yıldırım et al. (in press), who reported a single-factor structure with satisfactory goodness-of-fit indices, (*χ*^2^ [df = 27 = 222.77, *p* < 0.001, IFI = 0.94, TLI = 0.92, CFI = 0.94, RMSEA = 0.08, and SRMR = 0.04)]. In this study, the Cronbach’s alpha for the PSS was 0.82.

#### Perceived social support

Perceived social support was assessed using the Brief Perceived Social Support Questionnaire (BPSSQ) (Kliem et al., 2015), which measures individual differences in perceived social support at a general level. The BPSSQ consists of 6 items, with participants rating each on a 5-point Likert-type scale ranging from 1 (not true at all) to 5 (very true). The total score is calculated by summing the items, with higher scores indicating greater perceived social support. Yıldırım and Tanrıverdi (2021) validated the Turkish version of the scale, demonstrating strong reliability and validity. In this study, the Cronbach’s alpha for the BPSSQ was 0.81.

#### Psychological flexibility

Psychological flexibility was evaluated by utilising the Psy-Flex Scale (Gloster et al., 2021), which is an unidimensional self-report tool designed to assess contextually sensitive psychological flexibility. It consists of six items and participants respond on a 5-point Likert scale ranging from 1 (very seldom) to 5 (very often). Yildirim and Aziz (2023) validated the Turkish version by confirming its reliability and validity. In this study, the Cronbach’s alpha for the Psy-Flex was 0.74.

#### Psychological wellbeing

Psychological wellbeing was measured employing the Riverside Eudaimonia Scale (RES) (Margolis et al., 2022), which is a 5-item self-report measure designed to assess perceptions of eudaimonic wellbeing across various dimensions emphasised in philosophical discussions of eudaimonia. Each item is rated on a 7-point Likert scale from 1 (strongly disagree) to 7 (strongly agree), with higher scores indicating greater eudaimonic wellbeing. The Turkish version demonstrated good evidence of reliability and validity (Yıldırım and Çağış, 2023). In the current study, the RES showed a Cronbach’s alpha of 0.75.

#### Procedure

This research was part of a broader study focused on validating a positive solitude measure and its relationship with psychosocial variables, with distinct hypotheses beyond those reported elsewhere. Data were collected through a web-based survey using a snowball sampling method. Informed consent to participate was obtained from all of the participants in the study. Participants were fully briefed on the study’s purpose and their rights before, during, and after participation. Anonymity and confidentiality were ensured, and participation was entirely voluntary, with no compensation provided. The order of measures was consistent for all participants. The study followed the guidelines of the Helsinki Declaration, and Institutional Review Board (IRB) approval was secured prior to data collection.

#### Statistical analyses

The questionnaire was designed so that all items required a response before submission. Therefore, no participants were able to skip questions, and the dataset contained no missing values. As a result, the questionnaire completion rate was 100%, and no cases were excluded from the analysis. In Figure 1, perceived social support and psychological flexibility are modelled as mediators (*M*) in the relationship between positive solitude (*X*) and psychological wellbeing (*Y*). To assess the mediation effect, we used the bootstrapping method with 5,000 resamples, which allows for an estimation of indirect effects (Hayes, 2009). This approach resamples the dataset multiple times to generate simulated samples, increasing the accuracy of mediation analysis. All analyses were performed using IBM SPSS Statistics version 27.0 for Windows, along with the PROCESS macro.

## Results

### Descriptive statistics and correlation analysis

Table 1 presents the descriptive statistics, including mean, standard deviation, normality tests, correlation results, and internal reliability estimates for the study variables. Skewness and kurtosis values ranged from −0.50 to 0.37, indicating that all variables followed a normal distribution, adhering to the conventional guidelines for skewness and kurtosis values below |1| (Field, 2013). Correlation analysis revealed that positive solitude was significantly positively correlated with perceived social support, psychological flexibility, and psychological wellbeing. Also, perceived social support had a strong positive correlation with psychological flexibility and psychological wellbeing. Additionally, a significant positive relationship was found between psychological flexibility and psychological wellbeing.

**Table 1:** Descriptive statistics, reliability, and correlations between the study variables.

Variable	Descriptive statistics				Reliability	Correlations			
	Mean	SD	Skewness	Kurtosis	*α*	1	2	3	4
1. Positive solitude	32.62	5.99	−0.44	0.37	0.82	1			
2. Perceived social support	21.20	5.41	−0.50	−0.34	0.81	0.18^a^	1		
3. Psychological flexibility	18.96	4.28	−0.22	0.15	0.74	0.30^a^	0.31^a^	1	
4. Psychological wellbeing	25.33	6.00	−0.50	−0.13	0.75	0.36^a^	0.35^a^	0.40^a^	1

a Correlation is significant at the 0.01 level (2-tailed).

### Mediation analysis

The mediation analysis (see Tables 2, 3, and Figure 1) revealed that positive solitude had a significant positive effect on perceived social support (*β* = 0.16, *p* < 0.001) and psychological flexibility (*β* = 0.30, *p* < 0.001). Positive solitude accounted for 3% of the variance in perceived social support and 9% in psychological flexibility. Additionally, positive solitude (*β* = 0.24, *p* < 0.001), perceived social support (*β* = 0.23, *p* < 0.001), and psychological flexibility (*β* = 0.25, *p* < 0.001) significantly predicted psychological wellbeing, explaining 27% of the variance in psychological wellbeing. The indirect effects of positive solitude on psychological wellbeing through perceived social support (effect = 0.04, 95% CI [0.02, 0.06]) and psychological flexibility (effect = 0.07, 95% CI [0.05, 0.10]) were also significant, indicating that perceived social support and psychological flexibility partially mediate the impact of positive solitude on psychological wellbeing.

**Table 2:** Unstandardized coefficients for the mediation model.

Antecedent	Consequent			
	Coeff.	*SE*	*t*	*p*
	*M_1_* (Perceived social support)			
*X* (positive solitude)	0.16	0.03	5.79	<0.001
Constant	16.03	0.91	17.67	<0.001
	*R*^2^ = 0.03; *F* = 33.49; *p* < 0.001			
	*M_2_* (Psychological flexibility)			
*X* (positive solitude)	0.21	0.02	10.04	<0.001
Constant	12.09	0.70	17.35	<0.001
	*R*^2^ = 0.09; *F* = 100.69; *p* < 0.001			
	*Y* (Psychological wellbeing)			
*X* (positive solitude)	0.24	0.03	8.78	<0.001
*M_1_* (perceived social support)	0.26	0.03	8.31	<0.001
*M_2_* (psychological flexibility)	0.35	0.04	8.81	<0.001
Constant	5.27	1.06	4.99	<0.001
	*R*^2^ = 0.27; *F* = 128.92; *p* < 0.001			

*SE* = standard error, Coeff = unstandardized coefficient, *X* = independent variable, *M* = mediator variable, *Y* = dependent variable.

**Table 3:** Completely standardized indirect effects.

Path	Effect	*SE*	BootLLCI	BootULCI
Total indirect effect	0.12	0.02	0.08	0.15
Positive solitude –> Perceived social support –>Psychological wellbeing	0.04	0.01	0.02	0.06
Positive solitude –>Psychological flexibility –>Psychological wellbeing	0.07	0.01	0.05	0.10

Number of bootstrap samples for percentile bootstrap confidence intervals: 5,000.

## Discussion

Identifying factors that promote psychological wellbeing helps individuals function effectively. The results of this study showed that positive solitude had a positive association with perceived social support, psychological flexibility, and psychological wellbeing, which confirmed our first hypothesis. Adults who experienced positive solitude reported high perceived social support, psychological flexibility, and psychological wellbeing. Consistent with our results, the experience of solitude for pleasure supported the wellbeing of individuals (Samangooei et al., 2023). Emotion regulation skills may be positively affected by this situation, as the individual who can be alone with themselves has the space and time to regulate their emotions. Individuals with good emotion regulation skills also appear to be positively related to their wellbeing (Newman and Nezlek, 2021).

In addition, psychological inflexibility, which is the rigidity in an individual’s thoughts and behaviours to avoid unwanted emotional experiences, led to loneliness among young adults (Radyani et al., 2022). Emerson et al. (2021) revealed that friend support was negatively associated with loneliness. Since the individual knows he will receive social support if needed, he may prefer solitude as a source of renewal or autonomy rather than fear of being alone. Based on all this, wellbeing does not depend solely on external factors; it is also associated with the concept of positive solitude, which the individual establishes with himself, and that has positive benefits for him.

This study found that perceived social support and psychological flexibility were positively related to psychological wellbeing and confirmed our second hypothesis. Consistent with the social support and psychological flexibility literature, the findings of this study showed the relationship between perceived social support, psychological flexibility and psychological wellbeing. One study found that as friend support increased, individuals were less likely to experience psychological distress (Barstead et al., 2018). Another study revealed that individuals who perceived more social support had higher self-esteem and psychological wellbeing (Poudel et al., 2020). On the other hand, individuals who had the ability to adapt their psychological thoughts and behaviours according to the situation had better mental health outcomes (Young et al., 2022), which led to higher wellbeing (Pyszkowska and Rönnlund, 2021). These associations demonstrate that psychological flexibility not only reduces adverse effects but also improves psychological difficulties and wellbeing. Conservation of Resources Theory (COR Theory) states that individuals strive to obtain and protect valuable resources, and thatj resource loss increases psychological distress and reduces wellbeing (Hobfoll et al., 1990). Based on the above-mentioned research results, perceived social support and psychological flexibility, which are important for an individual’s mental health, can be expressed as resources. There appears to be a positive relationship between an individual’s perceived social support and psychological flexibility (Tindle et al., 2022). The concepts of perceived social support and psychological flexibility can mutually enhance one another and contribute to resource conservation, thereby increasing the individual’s wellbeing.

The SDT discussed in the introduction and the COR Theory discussed in this chapter are consistent with the process of meeting basic psychological needs and the pattern of protecting an individual’s psychological resources. This is because, as COR Theory states, the positive solitude that an individual may prefer due to their need for autonomy, and the social support they perceive due to their need for relatedness, function as valuable psychological resources that protect against stressors.

The current study also confirmed our third hypothesis by determining the mediating roles of perceived social support and psychological flexibility in the relationship between positive solitude and psychological wellbeing. Participants with high levels of positive solitude stated high perceived social support and high psychological flexibility, which in turn led to high psychological wellbeing. There are previous studies regarding the mediating roles of perceived social support and psychological flexibility. Perceived social support buffered the adverse effect of being alone on negative affective experiences (Fang et al., 2022). Psychological flexibility was an important mediating mechanism in the link between psychological resources and depression (Pellerin et al., 2022) and enhanced the positive effect of optimism on psychological wellbeing (Dalal and Singh, 2023). These studies demonstrated that perceived social support and psychological flexibility were significant drivers of high wellbeing. The present study suggests that perceived social support and psychological flexibility may strengthen the beneficial effect of positive solitude on psychological wellbeing.

The results of this study contribute to the existing literature on how psychological wellbeing can be improved in adults through positive solitude, perceived social support, and psychological flexibility. Wellbeing can differ in both quantity and associated factors between young and old adults (Röcke and Brose, 2013). Because of this fluctuation, supporting wellbeing throughout adulthood is important, and this study offers some practical implications. The experience of solitude, which encompasses intellectual and spiritual elements, offers physical and personal freedom and fosters inner authenticity and self-development (Fu, 2023). These positive effects in solitude may lead to wellbeing (Jiang et al., 2019). Therefore, encouraging adults to experience positive solitude may be beneficial for their wellbeing. In addition, desired solitude strengthens the perception of support by enabling individuals to develop a more harmonious and balanced relationship (Zambrano Garza et al., 2024). On the other hand, the tendency to respond to situations that facilitate the pursuit of valued goals for the individual contributes to wellbeing (Bi and Li, 2021). Thus, social support and psychological flexibility skill contents can be added to practices to increase the wellbeing of individuals.

On the other hand, collectivist values in Turkish culture significantly shape perceptions of positive solitude. This is because in collectivist cultures, individuals are seen as essential parts of social groups, such as family and friends. In this case, if an individual chooses to be alone, they may face negative stigma and social failure from these social groups. The idea that being alone could be a problem is one of the first reasons that comes to mind in this culture. Furthermore, social support plays a significant role in this culture. Individuals may believe they can cope with difficulties more easily because they have multiple options for receiving support during challenging times.

Despite the significant contributions of the present study, some limitations should be taken into consideration. The self-report data and cross-sectional design used in the study can be considered as restrictive elements in terms of the generalizability of the findings. Because of the cross-sectional design, these findings should be interpreted with caution and do not imply causal relationships. To overcome the limitations of self-report and cross-sectional analyses, it is recommended to conduct longitudinal and experimental studies using different data collection methods to better understand the supportive roles of social support and psychological flexibility. While the indirect effects were statistically significant, the relatively small effect sizes suggest that other important variables contribute to psychological wellbeing and should be explored in future research. In addition, our sample was composed of adults of a wide range of ages. Future studies may examine the tested model with a specific age group. Future research should examine alternative models, including moderation effects and different causal orderings of variables. The current research sample consists predominantly of young, female, and single individuals, limiting the generalizability of the findings to the Turkish adult population. Given that the sample was limited to individuals with internet access and specific social networks, the use of snowball sampling and web-based data collection may have introduced selection bias.

In conclusion, the present study emphasises that perceived social support and psychological flexibility are key points in the practices to be implemented to support the psychological wellbeing of adults. This study found that positive solitude was positively associated with perceived social support, psychological flexibility, and psychological wellbeing. Perceived social support and psychological flexibility had positive relationships with psychological wellbeing. Considering the effects of social support and psychological flexibility on the wellbeing of individuals, our results confirmed that perceived social support and psychological flexibility enhanced the positive effects of positive solitude, accordingly related to adults’ psychological wellbeing. The data suggest that perceived social support and psychological resilience can be key components for intervention programs that enhance wellbeing. However, to clarify the causal link between these variables and to establish concrete clinical protocols, priority should be given to experimental and longitudinal studies.

## Data Availability

The raw data supporting the conclusions of this article will be made available by the authors, without undue reservation.
